# The Many Alternative Faces of Macrophage Activation

**DOI:** 10.3389/fimmu.2015.00370

**Published:** 2015-07-22

**Authors:** David A. Hume

**Affiliations:** ^1^The Roslin Institute and Royal (Dick) School of Veterinary Studies, University of Edinburgh, Edinburgh, UK

**Keywords:** macrophage, transcriptomics, activation, colony-stimulating factor, lipopolysaccharide

## Abstract

Monocytes and macrophages provide the first line of defense against pathogens. They also initiate acquired immunity by processing and presenting antigens and provide the downstream effector functions. Analysis of large gene expression datasets from multiple cells and tissues reveals sets of genes that are co-regulated with the transcription factors that regulate them. In macrophages, the gene clusters include lineage-specific genes, interferon-responsive genes, early inflammatory genes, and genes required for endocytosis and lysosome function. Macrophages enter tissues and alter their function to deal with a wide range of challenges related to development and organogenesis, tissue injury, malignancy, sterile, or pathogenic inflammatory stimuli. These stimuli alter the gene expression to produce “activated macrophages” that are better equipped to eliminate the cause of their influx and to restore homeostasis. Activation or polarization states of macrophages have been classified as “classical” and “alternative” or M1 and M2. These proposed states of cells are not supported by large-scale transcriptomic data, including macrophage-associated signatures from large cancer tissue datasets, where the supposed markers do not correlate with other. Individual macrophage cells differ markedly from each other, and change their functions in response to doses and combinations of agonists and time. The most studied macrophage activation response is the transcriptional cascade initiated by the TLR4 agonist lipopolysaccharide. This response is reviewed herein. The network topology is conserved across species, but genes within the transcriptional network evolve rapidly and differ between mouse and human. There is also considerable divergence in the sets of target genes between mouse strains, between individuals, and in other species such as pigs. The deluge of complex information related to macrophage activation can be accessed with new analytical tools and new databases that provide access for the non-expert.

## The Cells of the Mononuclear Phagocyte System

The mononuclear phagocyte system (MPS) (1) is a family of professional phagocytes derived from hematopoietic progenitor cells under the influence of specific growth factors. They are the front line innate defense against pathogens, drive appropriate acquired immune responses, initiate inflammation, and promote resolution and repair. (2–7). The MPS has been viewed as a linear sequence from pluripotent progenitors, through committed myeloid progenitors shared with granulocytes, to promonocytes and blood monocytes, finally giving rise to tissue macrophages (2–7).

Challenges to the unified concept of an MPS have been discussed elsewhere (4, 5, 8). Some authors claim that macrophages, notably the microglia of the brain (9) and the epidermal macrophages (Langerhans cells) of the skin (10, 11) are seeded entirely during embryonic development and are renewed by local proliferation (12–14). A critical review of the experimental basis for this conclusion has been published elsewhere (8). Monocytes obviously can give rise to tissue macrophages, and tissue macrophages can also self-renew (15). An assessment of the relationship between the two mechanisms depends upon the interpretation of lineage-trace studies in mice that are open to criticism (8).

The abundance of monocytes and tissue macrophages is controlled through the macrophage colony-stimulating factor receptor (CSF1R), which is activated by two ligands, CSF1 and IL34 (6, 16–20). Treatment of mice with monoclonal antibody against CSF1R depletes most tissue macrophage populations in adult mice (21). Csf1r is itself a macrophage marker gene. The promoter of the mouse gene was used to generate transgenic *MacGreen* mice in which all of the tissue macrophages express an EGFP reporter gene (22). Myeloid-specific transgenes, using this and other tissue-restricted promoters, have been used in many studies of macrophage cell biology [reviewed in Ref. (23)].

## How Do We Define a Macrophage?

The network tool BioLayout *Express*^3D^ supports user-friendly visualization of co-expression relationships in large datasets (24, 25). We used this tool to identify co-regulated genes in the mouse BioGPS dataset (26) and in other mouse (27), human (28), and pig gene expression data (29). Macrophages as recognized by Metchnikoff are first and foremost professional phagocytes. To carry out that function, they must express all of the genes required to internalize particles and to degrade those particles in lysosomes. To identify those genes, we can compare the expression profiles of macrophages with cells that are not phagocytic. The underlying principal of guilt-by-association was recently extended to RNAseq data including long non-coding RNAs in the networks (30). Within the BioGPS dataset, different hematopoietic cell types expressed lineage-specific transcription factors alongside known lineage-specific genes (26). One obvious functional set of genes shared by all of the phagocytes (including bone-resorbing osteoclasts) contains the components of lysosomes, such as vacuolar ATPase H^+^ pump and lysosomal hydrolases. The significant numbers of genes within this set that possess little associated annotation are therefore likely to also participate in phagocytosis. The phagocyte-enriched gene set also contains a set of transcription factors that are linked in turn to their likely binding sites in phagocyte-restricted promoters. For example, the promoters of these phagocyte-restricted genes contain purine-rich motifs (binding sites for the macrophage-specific transcription factor, PU.1) alongside the motifs bound by basic helix–loop–helix transcription factors of the microphthalmia transcription factor family; MITF, TFEB, TFEC, and TFE3. All four MITF family members are expressed in macrophages. TFEC is a macrophage-specific transcription factor and itself a PU.1 target gene (31). MITF interacts both physically and genetically with PU.1 (32) and is able to transactivate the promoter of the ACP5 lysosomal enzyme (32). So, taken together, the data provide a picture of the transcriptional network required to become a professional phagocyte and provide a signature that distinguishes macrophages from other cell types.

The most controversial distinction in this respect is between macrophages and dendritic cells (DC). In the minds of some, the term “dendritic cell” has become almost synonymous with antigen-presenting cell (APC). However, the term clearly groups together cells of quite different function and origin (5, 8, 33–35). Active APC grow *in vitro* by cultivation of monocytes (in humans) or bone marrow cells (in mice) in GM-CSF. These cultured-derived APCs are quite distinct from “classical DC” or “conventional DC,” which express the growth factor receptor, Flt3, and differentiate in response to Flt3L *in vitro* and *in vivo* (35–37). The immunological genome consortium (ImmGen) produced datasets comparing mouse macrophages and “DC” from multiple sources. They claimed to have identified a DC signature (38) as well as markers (*Cd64* and *Mertk*) that defined macrophages as a separate cell type (39). They based the analysis upon comparison of DC with “prototypical” macrophages from peritoneum, lung, brain (microglia), and splenic red pulp that were known already to lack class II MHC. We could not confirm the validity of the signatures of either cell type (34). Instead, the APC could be separated based upon their expression of the phagocyte gene cluster. The microarray platforms are now sufficiently reproducible and the processing methods standardized, that one can actually integrate datasets from independent laboratories, so it is not necessary to perform all studies in one laboratory. In microarray datasets from mouse and human (27, 28), cells annotated as DC form two distinct clusters. Cells grown in GM-CSF segregate with the macrophages in a separate class from classical “DC” isolated from lymphoid organs. The FANTOM5 consortium produced data that enable promoter-based clustering of co-expression networks, which support the same distinction between two different classes of APC (40, 41). The same data supported a study of the process of macrophage differentiation from pluripotent progenitors through monocytes to macrophages (40, 41). Overall, all the data are consistent with the original definition of the classical DC by Steinman and Cohn; which *“unlike macrophages, do not appear to engage in active endocytosis”* (42). So, I take the view that antigen presentation is a function, not a cell type, and prefer to restrict the use of the term DC to APCs that depend up Flt3L.

## Macrophage Activation

Macrophages are abundant in every organ of the body, but each tissue macrophage population is distinct (34). For example, microglia, the macrophages of the brain, are quite different from blood monocytes and tissue macrophages isolated from other locations (43). Indeed, as discussed above, a *Csf1r*-EGFP transgene has been used as a marker for all tissue macrophages (22). In most organs, there are so many macrophages that we can infer the macrophage signature from analysis of total mRNA. In pigs, for example, we profiled lung macrophages, blood monocytes, and bone marrow-derived macrophages alongside most major organs (29). Alveolar macrophages expressed many different C-type lectin receptors, presumably to recognize and engulf inhaled microorganisms. In mRNA from the wall of the gut, macrophage-specific transcripts derived from the abundant lamina propria macrophage population were easily detected, but the C-type lectins were undetectable. We infer that recognition of gut luminal microorganisms by macrophages in the lamina propria would be undesirable.

Macrophages change their gene expression in response to many different stimuli. Aside from driving tissue-resident macrophage heterogeneity, this plasticity enables an appropriate response to pathogen challenge. The term “activation” applied originally to recruited macrophages that acquired tumoricidal and microbicidal activity. Activation occurred in two phases, a priming event initiated by T cell products and a triggering event generated by lipopolysaccharide (LPS) (44, 45). The activation states in recruited macrophages have since been classified as M1 and M2, or classically activated and alternatively activated (3, 46–49). M1 and M2 activation of macrophages is associated with Th1 and Th2 lymphocytes, and in turn with interferon-gamma (IFNγ) (45) and interleukin 4 (IL4) as the mediator. Classical or M1 activation mediates defense against bacterial pathogens. Classically activated macrophages also express class II MHC, and present antigen to T lymphocytes. M2 or alternatively activated macrophages are produced in parasite infections and tumors and express the targets of IL4 signaling (50–52). The house of cards built on this foundation included the identification of M2 markers, such as Arg1, Retnla, Chi3l3 (53).

The nomenclature has become increasingly confused. Macrophages grown in GM-CSF are said to be M1-like (when they are not called DC), and those grown in CSF1 to be M2-like (50). Different activation states require distinct transcription factors, such as the IRF transcription factor family; STAT1 and IRF5 induce M1 polarization where STAT6 and IRF4 interact to polarize toward M2 (50). The quite different response to macrophages to toll-like receptor agonists such as LPS (54–59) has also been called an M1 response. This response involves the sequential induction of hundreds of genes and includes a complex intrinsic and inducible feedback control network [see below, Ref. (60)]. We recently compared responses to IFNγ and IFNβ and the response to LPS (61). There was limited overlap. Interestingly, the LPS target genes differ in their dose-responsiveness, so even the response to a single agent cannot be classified.

The problem with nomenclature in macrophage activation prompted a group of researchers in the field to try and establish a common framework (53). They noted that the origins of the M1/M2 concept came from the significant biases in macrophage polarization between C57Bl/6 (M1) and BALB/c (M2) mice. Macrophages from different mouse strains differ substantially in their gene expression profiles (58, 61, 62). As discussed further below, these differences can be related to gain and loss of transcription factor-binding sites (58) and are also associated with allele-specific DNA methylation patterns in macrophages from F1 crosses between strains (63). Some of the sequence changes between strains produce clear strain-specific variation that is completely idiosyncratic. For example, the C57Bl/6 mouse has a deficiency in cathepsin E expression, due to loss of a PU.1 site in the promoter, and is therefore deficient in antigen processing (64).

The attempt by Murray et al. (53) to establish a consensus view of activation nomenclature still favors the use of combinations of markers to describe macrophage polarization states. Unfortunately, there is absolutely no support for the usefulness of markers in genome-scale data. There is no set of genes that is co-expressed in large datasets that could be used as a signature of a macrophage activation state. Just as DC has been conflated with APC, the “alternatively activated” macrophage is often a synonym for IL4-stimulated. For example, the implied role of “alternatively activated macrophages” in adaptive thermogenesis was no more than a study of the function of IL4-inducible target genes (65). Interestingly, the macrophage-restricted transcription factor, TFEC, appears to have a specific function in the inducible expression of a subset of IL4-response genes (66). The M1/M2 concept does not translate well across species. The gene expression profiles of “activated” mouse, pig, and human macrophages are quite distinct (67), with the pig more human-like (68, 69). Comparative analysis of IL4-stimulated mouse and human macrophages identified transglutaminase 2 as a “conserved M2 marker” (52). However, this did not appear in any co-expression clusters from large mouse or human datasets.

As discussed elsewhere (70), I personally support Mosser and Edwards (71) suggestion that macrophage activation at a population level at least is analogous to a color wheel. The extremes of polarization of macrophage function may be seen as red, blue, or yellow, but macrophages exist in every hue and intensity in between. Xue et al. (72) confirmed the spectrum model based upon analysis of human monocyte-derived macrophages grown in CSF1 or GM-CSF and exposed to stimuli including IFNγ, IFNβ, IL4/IL13, IL10, glucocorticoids, TLR agonists, TNF, and prostaglandin. While there was some separation between IFNγ (M1) and IL4 (M2) directed states, the transcriptional response could be divided into 49 distinct co-expression clusters with 27–884 genes per module. Even these modules are clearly a simplification based upon synchronous stimulation with individual agents or combinations. As they enter an inflammatory site *in vivo*, macrophages are exposed to a complex gemisch of signals and the cellular phenotype changes with time. As we move toward single cell profiling in diseased tissues, we will inevitably come to the conclusion that every macrophage is unique.

The tumor-associated macrophage has been considered the archetype of the M2 phenotype (7, 73). Because the stromal component of a tumor biopsy is not uniform, each sample differs in its relative macrophage content. For that reason, we can extract a common macrophage co-expression signature from the expression profile of solid tumors (74). This signature includes macrophage markers, CD68, CD14, and CSF1R, many lysosomal genes, class II MHC, co-stimulatory molecules, and markers of both the M1 and M2 phenotypes. There is a separate set of interferon-inducible genes in many datasets, including tumors (74) implying that these genes are regulated independently of macrophage content. As noted above, we have defined the interferon responses in mice (61) and the interferon signature in the response of pig macrophages to LPS, which differs between breeds (69, 75). Another study (76) compared the responses of human monocytes from several hundred donors to IFNg and LPS. Each stimulus produced a stereotypical response (77), but there was also variation in expression of individual genes, with eQTLs associated with the majority of loci. The authors (76) identified an SNP associated with the level of expression of interferon (IFNβ1) after 2 h; linked in trans to expression of IFN responsive genes after 24 h of LPS treatment. The targets include many anti-viral effectors including IFITM2 and IFITM3, implicated in genetic susceptibility to influenza (78). On this basis, the variation in IFN-response genes between patient samples in many large datasets probably reflects the underlying genetic predisposition rather than the disease state. The interferon signature is present in gene expression profiles in tissues and blood in human infections and chronic inflammatory diseases, and implicated in severity, prognosis, and progression (78–82).

## Dynamic Networks in Macrophage Differentiation and Activation

Macrophage activation by any agonist alters gene expression radically during the transition from one steady state to another. Features of the regulatory cascade are exemplified by the detailed analysis of the response to LPS.

**Figure 1:**
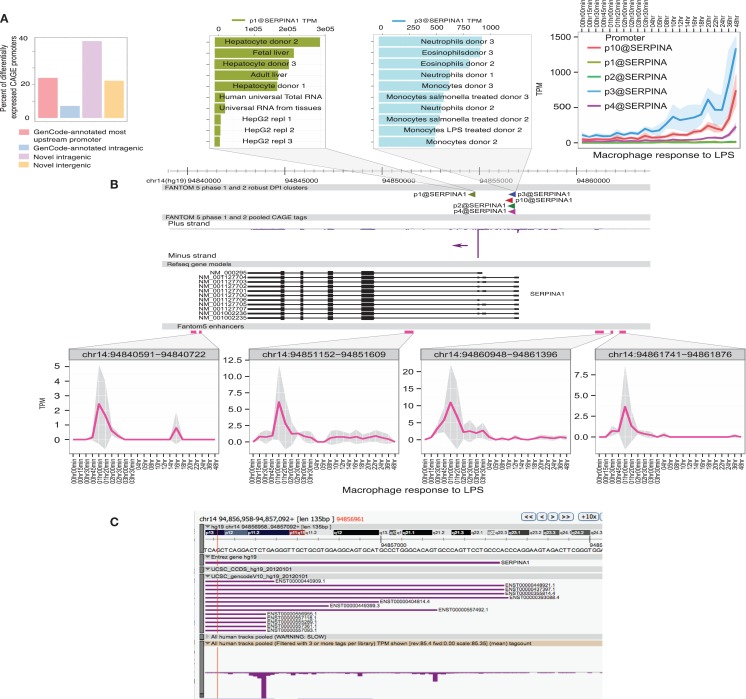
**Transcriptional regulation of *SERPINA1* in human macrophages**. The FANTOM5 analysis across hundreds of cells and tissues revealed the existence of multiple transcription start site (TSS) clusters in the vicinity of the SERPINA1 gene, as well as at least six enhancers in the genomic facility. At top left, **(A)** summarizes the fact that existing annotated upstream TSS in GenCode contributes only 20% of the TSS detected across the entire dataset. The majority of transcripts derive from two “intragenic” regions. The expanded genomic view above links the TSS to the expression profile. Note that the most abundant TSS, p1@serpinA1, was detected most highly in liver and in primary hepatocyte libraries, and much less in the relatively de-differentiated HepG2 cells. The second most abundant TSS, p3@serpinA1, was constitutively active in granulocytes. At top right, we see that three of the distal promoters were induced by LPS in human monocyte-derived macrophages, starting around 3–4 h after stimulation. The lower part of the panel **(B)** shows the location, and the time course of induction, of four separate enhancers, upstream, downstream, and within the *SERPINA1* gene. **(C)** shows a close-up view of the distal TSS region on the ZENBU viewer, showing that the TSS identified by CAGE do align with known transcripts, but none supports the most distal 5′ end annotated by Entrez Gene. The primary data summarized in this image are derived from Arner et al. (100) and may be freely downloaded. The CAGE data were extracted and the diagram produced by Albin Sandelin and Erik Arner. The original data were produced in collaboration with Kenneth Baillie.

**Figure 2:**
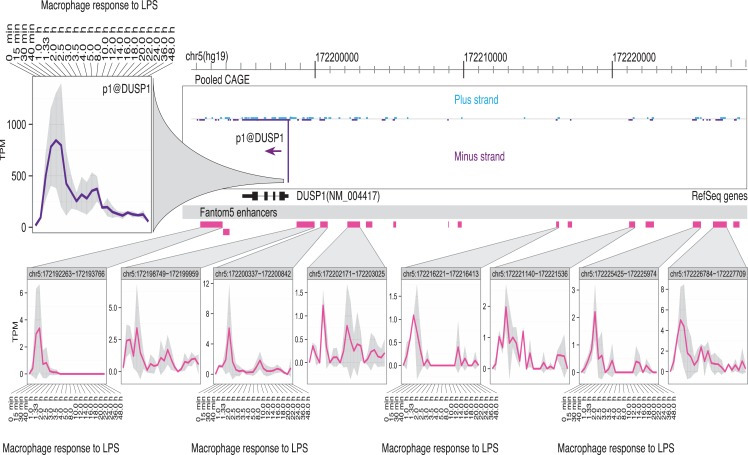
**Transcriptional regulation of *DUSP1* in human macrophages**. The FANTOM5 analysis across hundreds of cells and tissues revealed the existence of multiple transcription start site (TSS) clusters in the vicinity of a single dominant TSS for the *DUSP1* gene, with a classical TATA-box architecture typical of highly inducible genes, as well as at least 14 enhancers in the genomic facility. Upper left panel shows the induction of transcription from this TSS when human monocyte-derived macrophages were treated with LPS. The lower part of the panel illustrates the fact that at least eight of these enhancers showed detectable activation of production of eRNAs detected by CAGE tags. Note that the enhancers were activated around 1–1.5 h, where the peak of DUSP1 transcripts was detected around 2.5 h. The primary data summarized in this image are derived from Arner et al. (100) and may be freely downloaded. The CAGE data were extracted and the diagram produced by Albin Sandelin and Erik Arner. The original data were produced in collaboration with Kenneth Baillie.

(1) There is a sequential cascade of gene regulation. This cascade has been analyzed in great detail in mouse bone marrow-derived macrophages (54, 55, 61). The early response genes, including classical inflammatory cytokines, such as TNFα, are controlled at the level of transcription elongation from poised RNA pol II complexes (83, 84). Later response genes are regulated by autocrine factors, including TNF and IFNβ1, and by numerous induced and repressed transcription factors regulated downstream of the initial signal (54–59).
(2) The response of individual target genes to LPS requires the interaction between promoters and poised enhancers. In the macrophage lineage, a critical “pioneer” factor that binds to these elements is PU.1. PU.1 mRNA is expressed in both myeloid and lymphoid cells, but the level of PU.1 protein in the nucleus is vastly higher in macrophages (85, 86). These high levels permit PU.1 binding to relatively low affinity sites, to support both constitutive and stimulus-inducible transcriptional regulation (56, 87–90). Macrophage-specific transcriptional enhancers involve the interaction between PU.1 and multiple other transcription factors, including the MITF family, IRF8, AP1, Klf, and Rel (NFκB) family members (56, 87–90). These factors are themselves regulated by LPS. A recent study separated the characteristics of IRF8 binding to constitutive and inducible enhancers. IRF8–PU.1 cooperated to support basal expression of macrophage-specific genes. Conversely, binding of IRF8 in the vicinity of inducible genes required cooperation with other inducible transcription factors, including AP1, IRF, and STAT family members (91).
(3) The numbers and magnitudes of regulation/expression of induced genes are balanced by transcriptional repression of other genes. Analysis of CAGE tag libraries [genome-scale 5′-RACE (92, 93)] revealed that individual transcript abundance follows a power law relationship with a constant exponent (94). So, gene induction of some genes must be balanced by repression of others. In LPS-stimulated macrophages, the repressed genes include transcriptional repressors that can block the response to LPS, as well as genes involved in other pathways. For example, LPS produced growth arrest in macrophages, so the LPS-downregulated genes include those required for the response to the growth factor, CSF1, and the cell cycle (61).
(4) The response to LPS is self-limiting even in the continued presence of the agonist. The LPS-inducible genes are numerous additional feedback-regulators (60, 61, 95) described as “inflammation suppressor genes” (60, 61). They include many inducible splice variants that encode competitive inhibitors of signaling (96). These inducible feedback regulators act at every level of the TLR signaling cascade. The intuitive explanation for this complex feedback regulation is that they represent an “effective self-control mechanism to ensure that sufficient levels of innate and adaptive immune response are induced to combat pathogens but to avoid “over-heating” the system once this has been achieved” (95). However, the feedback is not robust, since even heterozygous knockouts of many of the feedback repressors produce severe pathology, and many are polymorphic within populations (60). The alternative view is that the complex feedback regulation produces population heterogeneity between macrophages and between individuals. In essence, when dealing with an ever-changing pathogen landscape, it is desirable not to have all the macrophage eggs in one basket.
(5) Enhancer activation precedes the activation of specific target promoters. The activation of enhancers can be detected in LPS-stimulated macrophages through the production of so-called eRNAs, transcribed by RNApolII (97, 98). The use of genome-scale 5′-RACE (CAGE) by the FANTOM5 consortium enabled the quantitative analysis of these eRNAs, which are generally transcribed equally in both directions from active enhancers, with transcription start sites (TSS) around 150–200 bp apart. The same technology detects active promoters in the genomic vicinity, and one can infer the likely relationship between active promoters and enhancers that show the same expression pattern across a large cell and tissue atlas (99). This association is more powerful when one has access to detailed time course data. As part of the FANTOM5 project, we produced a detailed time course of the response of human monocyte-derived macrophages to LPS (100) (Ms in preparation). Figures 1 and 2 show examples of the power of this data. Figures 1A,B shows the well-studied *SERPINA1* (alpha-1-antitrypsin) locus. The data reveal that there are two promoters, one used in liver and the other in myeloid cells, as previously shown (101) and confirmed in the mouse (93). The *SERPINA1* gene is expressed constitutively in monocytes and granulocytes, repressed in monocyte-derived macrophages grown in CSF1, and strongly induced as a late-response genes upon addition of LPS. As shown in Figure 1A, the actual TSS in macrophages form a broad cluster, typical of myeloid promoters, around 50 bp upstream of the TSS originally identified, but downstream of the EntrezGene transcript. Within the promoter region, there are four copies of the CAGGAA core recognized by Ets family transcription factors, and it is likely that the induction of multiple members of the family by LPS, revealed in the same data set, contributes to regulation. As shown in Figure 1B, induction by LPS is preceded by increased transcription of multiple enhancers. The MAK kinase phosphatase, *DUSP1*, is one of the key inducible feedback regulators that is induced rapidly in response to LPS. As shown in Figure 2, the *DUSP1* gene was induced massively by LPS, with an initial peak at 2–2.5 h, and a secondary peak at 7–8 h. At least eight enhancers upstream and downstream of TSS were detectably induced by LPS, with peaks around 30–60 min prior to peak induction of *DUSP1* transcripts, and some evidence of secondary peaks.
(6) Population-level analyses assay the average behavior of cells in a population and obscure the massive underlying heterogeneity. At a single cell level, there is essentially bimodal variation; genes are either induced by LPS or they are not (102). One consequence is that the autocrine loops mediated by inducible cytokines are, in fact, paracrine and the response to LPS in closed systems, in cell culture or in defined inflammatory sites, can vary with cell density. Although some of this variation could arise through covariance of transcription factor expression, the major driver of heterogeneity is the intrinsic probabilistic nature of transcriptional regulation (103). Variation occurs even at the single allele level. Indeed, the LPS receptor, *TLR4*, is expressed from only one allele in individual cells, with an allele-counting mechanism similar to that of the X chromosome (104). This finding explains the semidominance of the *Tlr4* mutation in C3H/HeJ mice, since in heterozygotes, 50% of cells express the non-functional protein. When one allele is deleted, all cells express *Tlr4*. Furthermore, because of the complex feedback loops in stimulated cells, in which LPS rapidly induces inhibitors that block signaling and degrade induced mRNAs and proteins (60), individual cells show an oscillating response with relatively short time frames, eventually reaching a new steady state (105).

The signaling pathway from the LPS receptor, TLR4, leading to transcriptional activation, has been reviewed in detail by others (106, 107). The target genes of the signaling cascade are bound by a set of regulated transcription factors. The most obvious, of course, are NFkB and the IRFs, mentioned above. Genome-scale analysis revealed others. For example, motif over-representation among LPS-inducible promoters identified ATF3 as a novel inducible feedback regulator of the response to LPS (54). They also identified the stress response factors, NRF1 and NRF2, as candidate regulators, based upon the presence of binding site motifs in active promoters (55). We compared the motif over-representation in the promoters of LPS-inducible macrophage genes in humans and mice (67). The motifs over-represented in the promoters, and their relative over-representation, was conserved suggesting that in both species the promoters sample a common transcriptional milieu. However, there was a little conservation of individual elements between the species. So, the gain and loss of individual motifs, including the TATA box, is a significant driver of evolution, and few individual motifs/binding sites have indispensible functions. Those data also support the model in which each transcription factor-binding site contributes independently to the probability of transcription (108). But the “big names” of transcription are not the only players in the LPS-inducible transcriptional network. Around two-thirds of all transcription factors can be detected in primary macrophages, and they form a scale-free or small-world interaction network (55). Amit et al. (109) disrupted the candidate regulators using lentiviral transduction of short hairpin RNAs and identified many novel regulators, including circadian clock genes, that could not inferred from motif analysis.

Transcriptional network analysis reached a pinnacle in a study of a model system of human macrophage differentiation (110). Nearly half of all transcription factors were detected at some time point during differentiation and around 200 were dynamically regulated. Although the well-known macrophage lineage marker, PU.1, was crucial to differentiation, subsets of macrophage-expressed genes required different combinations of transcriptional regulators. Many transcription factors were rapidly repressed during the differentiation of THP-1 cells. Fifty-two of these factors were artificially repressed using siRNA. The knockdowns of myb, HoxA9, CEBPG, GFI1, CEBPA, FLI1, and MLLT3 were each sufficient to cause partial differentiation of the THP-1 cells. Additional double knockdowns proved that each contributed independently to the differentiation process. Several of these factors have well-documented repressive roles. The function of myb, in particular, correlates with its known downregulation as progenitor cells differentiate and exit the cell cycle^1^, and its ability to repress macrophage differentiation and to directly repress csf1r transcription (111). Myb is most likely also a direct repressor of PU.1 expression, since PU.1 was rapidly induced upon myb knockdown (110).

## Databases, Web Sites, and The Future

BioGPS is one example of a new era of emerging user-friendly portals (see text footnote 1) that enable the analysis of complex transcriptomic data. Although we disagree with the published analysis, the gene expression data produced by the ImmGen is an important resource for mouse functional genomics^2^. The web site, www.macrophages.com, was established as a community web site for sharing access to macrophage-related genomic and other information including the promoter-related datasets arising from the FANTOM projects (112). The site provides access to web-start versions of the Biolayout analysis of large macrophage datasets. The FANTOM5 consortium produced a comprehensive overview of the human and mouse promoterome, including mouse and human mononuclear phagocytes and other blood cell types analyzed in hundreds of different states, accessible through a convenient portal^3^. We developed a specialized portal for the myeloid data at www.myeloidome.roslin.ed.ac.uk. The PrESSTo system provides an intuitive promoter and enhancer selection interface on top of the FANTOM data. It permits the selection of promoters expressed in specific sets of tissues or cells based on slider thresholding, where the number of promoter adhering to a given combination of expression thresholds for cells or tissue can be updated in real time^4^. With completed genomes, comparable datasets are becoming available species such as the domestic pig, chickens, sheep, and cattle. As we have already seen, mice are not small humans when it comes to macrophage biology. Studies of the evolution of macrophage transcriptional networks will also reveal the points of weakness that are exploited by pathogens in specific species, and which may also be the points for therapeutic intervention.

## Conflict of Interest Statement

The author declares that the research was conducted in the absence of any commercial or financial relationships that could be construed as a potential conflict of interest.

## Footnotes

^1^http://www.biogps.org

^2^http://www.immgen.org

^3^http://fantom.gsc.riken.jp/zenbu/

^4^http://pressto.binf.ku.dk/
